# Mental health in medical, dental and pharmacist students: a cross-sectional study

**DOI:** 10.1192/j.eurpsy.2022.1511

**Published:** 2022-09-01

**Authors:** A. Frajerman, B. Chaumette, M.-O. Krebs, Y. Morvan

**Affiliations:** 1 University of Paris, INSERM, U1266 Institute Of Psychiatry And Neuroscience Of Paris, PARIS, France; 2 GHU Paris Psychiatrie et Neurosciences, Pepit, Paris, France; 3 McGill University, Department Of Psychiatry,, Montréal, Canada; 4 GHU Paris Psychiatrie et Neurosciences, Pepit, paris, France; 5 Université Paris Nanterre, Ufr Spse, Laboratoire Clipsyd, Nanterre, France; 6 Inserm, U1018, Cesp, Paris, France

**Keywords:** Anxiety, Depression, burnout, students

## Abstract

**Introduction:**

Health student’s mental health is considered a public health issue that dramatically increased with COVID 19’s pandemic. However, very few studies assessed the prevalence of mental health in medical, pharmacist, and dental students.

**Objectives:**

Our goal was to assess mental health in health students from the same university one year after pandemic’s beginning and look at for associated factors.

**Methods:**

An online survey was realized in Paris university which has the 3 specialties (medicine, pharmacy, and dental). We used the Hospitalization Anxiety and Depression scale, Composite International Diagnostic Interview - Short Form questionnaire, Maslach Burnout Inventory (with 2 versions (Student survey and Human Services Survey). We also asked for 12 months of suicidal ideation, humiliation, sexual harassment, and sexual aggression. We did multivariable logistic regression analyses to identify Major Depressive Episode (MDE) associated factors.

**Results:**

We included 1925 students: 95 dental, 233 pharmacists, 541 medical preclinic, 587 medical clinic and 469 residents. Overall prevalence of 7- days anxiety symptoms, 7- days depressive symptoms, 12-month MDE, 12-month suicidal ideation, humiliation, sexual harassment and sexual aggression were 55%, 23%, 26%, 19%, 19%, 22% and 6% respectively. There were significative differences between groups for anxiety and depressive symptoms and MDE (p<0.001 for all). Associated factors to MDE in multivariable logistic regression were humiliation (OR=1.71, IC95[1.28-2.28]), sexual harassment (OR=1.60, IC95[1.19-2.16]), sexual abuse (OR=1.65, IC95[1.04-2.60])) and moderate (OR=1.49, IC95[1.17-1.90]) or important (OR=2.32, IC95[1.68-3.20]) subjective financial difficulties.

**Conclusions:**

Health student’s prevalence of psychiatric symptoms is significant, but it seems possible to intervene on several risk factors.

**Disclosure:**

No significant relationships.

